# Malignant transformation of parathyromatosis to parathyroid carcinoma with invasive growth and distant metastasis

**DOI:** 10.1530/EO-25-0024

**Published:** 2025-05-31

**Authors:** Chittari Venkata Harinarayan, Anugnya Premdas Ranjoalkar, Anisha Sawkar Tandon, Honey Ashok, Madhu Prashant

**Affiliations:** ^1^ Institute of Endocrinology, Diabetes, Thyroid and Osteoporosis Disorders, Sakra World Hospitals, Bangalore, Karnataka State, India; ^2^ Department of Medicine & Endocrinology, Saveetha Institute of Medical and Technical Sciences University, Saveetha Medical College, Chennai, India; ^3^ Department of Laboratory Medicine, Sakra World Hospitals, Bangalore, Karnataka State, India; ^4^ Department of Radiology, Sakra World Hospitals, Bangalore, Karnataka State, India; ^5^ Department of ENT, Head and Neck Surgery, Sakra World Hospitals, Bangalore, Karnataka State, India; ^6^ Interventional Radiology, Sakra World Hospitals, Bangalore, Karnataka State, India

**Keywords:** primary hyperparathyroidism, parathyromatosis, parathyroid carcinoma

## Abstract

Parathyroid carcinoma (PC) is a rare endocrine malignancy. It accounts for about 1% of all primary hyperparathyroidism (PHPT) cases and 0.005% of all malignancies. PC presents with severe hypercalcemia, often refractory to standard medical treatment, which is crucial for improving patient outcomes. Differentiating PC from atypical parathyroid adenomas and parathyromatosis can be challenging. PC is diagnosed by angio-invasion, lymphatic and perineural invasion, and local malignant invasion with regional and distant metastasis. CD31 positivity on immunohistochemistry, indicating tumor cells within blood vessels in the pseudocapsule (vascular invasion), and a Ki-67% proliferative index of 8% confirm the diagnosis. We describe a 67-year-old man who presented with neck swelling, abdominal pain, and weight loss. Five years prior, he underwent a left inferior parathyroidectomy for a mass, which histological examination identified as clear cell parathyroid adenoma. Three years post-parathyroidectomy, he developed recurrent left-sided neck swelling. Magnetic resonance imaging revealed a lesion with mediastinal extension but no lymph node metastasis. During surgery, the lesion was adherent to the common carotid artery, and histopathological examination confirmed parathyromatosis. Recently, he presented again with new-onset neck swelling, abdominal pain, and weight loss. Biochemical investigations confirmed primary hyperparathyroidism. Invasive growth into surrounding tissues, blood vessels, and nerves, along with lymph node involvement and lung metastasis, with histopathology showing CD31 positivity and a Ki-67% proliferative index of 8%, confirmed the diagnosis of PC. As the tumor was inoperable, hypercalcemia was managed with cinacalcet, resulting in a decline in serum calcium levels.

**Learning points:**

- Parathyroid carcinoma is one of the rarest malignancies.
- Parathyromatosis is parathyroid tissue displaced in the neck and mediastinum due to prior surgery or aberrant embryonic development.
- The diagnosis of parathyroid carcinoma on histopathology is based on:
  - Local invasion into adjacent structures, such as adipose tissues/skeletal muscle bundles.
  - Loco-regional invasion–lymphatic (D240 IHC+), angio-invasion (CD31 IHC+), and perineural invasion.
  - Nuclear pleomorphism, hyperchromatic nuclei, and increased mitosis (high Ki-67 proliferative index of >5%).
  - Regional and distant metastasis in the lungs and other organs.
- Management of severe hypercalcemia is crucial for improving patient outcomes.

## Background

Parathyroid carcinoma (PC) is a rare endocrine malignancy. It accounts for about 1% of all primary hyperparathyroidism (PHPT) cases and 0.005% of all malignancies. PC affects both genders, typically between 45 and 55 years of age, and occurs sporadically. Less frequently, it is associated with hyperparathyroidism-jaw syndrome and, rarely, with MEN type 1 or type 2A syndromes (Erickson *et al.* 2022). Its clinical and biochemical features overlap with benign causes of PHPT, often leading to delayed diagnosis. PC presents with severe hypercalcemia, which is usually refractory to standard medical treatment, a key factor in improving patient outcomes.

Distinguishing between benign and malignant parathyroid lesions is histologically challenging due to their nonspecific and equivocal features. Differentiating PC from atypical parathyroid adenomas (PA) and parathyromatosis (PM) poses a diagnostic challenge (Schulte *et al.* 2021). PM consists of parathyroid tissue displaced in the neck or mediastinum, usually due to prior surgery or, rarely, aberrant embryonic development.

The WHO classification of endocrine organ tumors defines PA as a lesion that exhibits some features of PC but lacks unequivocal invasive growth. PC, however, is characterized by angio-invasion, lymphatic and perineural invasion, and local malignant invasion with regional and distant metastasis (Erickson *et al.* 2022). In contrast, PA and PM do not exhibit distant metastasis.

We present a novel case of malignant transformation from parathyroid adenoma to PM and ultimately to PC with invasive growth and distant metastasis. The rapid lesion growth, invading the trachea, esophagus, and carotid blood vessels, posed a diagnostic challenge, while life-threatening hypercalcemia proved difficult to manage.

## Case presentation

The clinical, biochemical, imaging, and surgical details are described in Supplementary Table 1 (see section on Supplementary materials given at the end of the article).

A 67-year-old man presented with neck swelling, abdominal pain, and weight loss. Five years earlier, he underwent a left inferior parathyroidectomy for a mass (6.5 × 5.6 × 4.9 cm). Histological examination revealed an encapsulated mass of clear cells with round nuclei and moderate cytoplasm, without atypical cells, mitosis, or vascular or capsular invasion. Three years post-parathyroidectomy, he developed recurrent left-sided neck swelling. Magnetic resonance imaging (MRI) showed a 5.1 × 8.1 × 8.5 cm lesion with mediastinal extension but no lymph node metastasis. During surgery, the lesion was adherent to the lower common carotid artery. Histopathological examination revealed unencapsulated nodules of varying sizes with smooth edges within the skeletal muscle tissue. The nodules contained uniform cells with clear-to-amphophilic cytoplasm and small dark nuclei, with a mitotic count of 1–2 per 50 high-power fields, consistent with PM.

**Figure 1:**
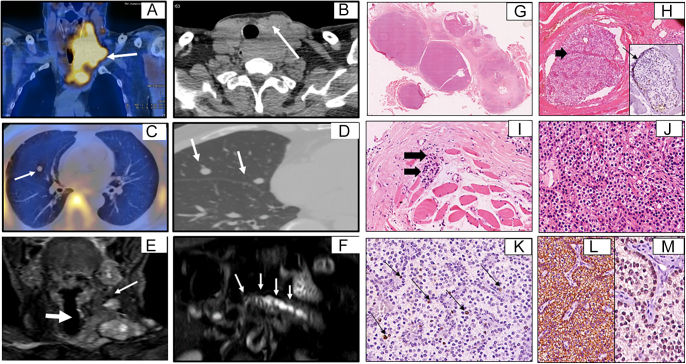
(A, B, C, D, E, F) C11-choline positron emission tomography-computed tomography (PET-CT). Diffuse soft tissue density mass lesion measuring 7 × 5.2 × 9.8 cm showing increased uptake on choline PET-CT in the location of the left parathyroid, invading the left lobe of the thyroid gland, cervical esophagus, left lateral wall of the trachea, overlying skin, adjacent strap muscles, with mediastinal extension ((A) coronal PET-CT images, short white arrow; (B) axial CT at D1 level, white arrow). Nodular lung metastatic lesions seen on PET-CT images ((C) white arrows) and on CT scan of the chest ((D) short white arrow). Heterogeneous signal intensity of a left parathyroid mass lesion with local invasion, as well as cervical lymph nodes ((E) short white arrow). Axial T2W MRI at the level of the pancreas shows dilated pancreatic duct and parenchymal atrophy, in keeping with features of chronic pancreatitis ((F) short white arrows). (G, H, I, J, K, L, M) Histopathology of metastatic deposit. Hematoxylin and eosin (H&E) stained specimens of the lobules of the monotonous parathyroid tumor show focal infiltration into the adjacent adipose tissue and skeletal muscle bundles; 40× (G). Infiltration of the parathyroid tumor cells into the skeletal muscle bundles and evidence of vascular invasion (arrowhead) is confirmed on CD31 immunohistochemistry (IHC), H&E; 100× (H). Biopsy of the muscle deposit shows monotonous tumor cells infiltrating the skeletal muscle bundles, H&E; 100× (I). Tumor cells exhibit mild to moderate focal nuclear pleomorphism, occasional prominent nucleoli, and moderate amounts of cytoplasm, H&E; 400× (J). Percent Ki-67 positivity was up to 8% (arrow), IHC; 100×, MiB-1 (PathnSitu) (K). Chromogranin – diffuse and strong membranous and cytoplasmic staining of tumor cells, IHC; 100×, EP38 (PathnSitu) (L). PAX8 – moderate nuclear staining of tumor cells, IHC; 400X, MD50 (Master Diagnostica) (M). Negative results for TTF1, synaptophysin, and D240.

At a recent presentation, the patient had new-onset neck swelling, abdominal pain, and weight loss. His vital signs were normal, and his body mass index was 23 kg/m^2^. Physical examination revealed a firm, moderately enlarged swelling in the left thyroid region. Biochemical investigations confirmed primary hyperparathyroidism. The clinical, biochemical, imaging, and histopathological features are shown in Supplementary Table 1 and Fig. 1. Thyroid, adrenal, and liver function test results were unremarkable.

## Investigations

The clinical, biochemical, radiological, surgical, and histopathological details of the case are outlined in Supplementary Table 1. Other biochemical reports were: serum ionized calcium 1.55 mmol/L (N 1.12–1.32), lipase 516 IU/L (N 23–300), and amylase 147 IU/L (N 30–110).

Laboratory investigations confirmed primary hyperparathyroidism (PHPT) (Supplementary Table 1). The urine calcium/creatinine ratio was 0.07 (normal <0.02), phosphate excretion index was 1.17, and bone mineral density measurements were as follows: lumbar spine, 1.070/−1.3; left hip, 0.732/−2.6; and left forearm, 0.755/−2.5.

C11-choline positron emission tomography-computed tomography and MRI findings are shown in Fig. 1A, B, C, D, E, F. Nodular metastatic lesions were observed on a chest CT scan. MRI at the pancreatic level revealed a dilated pancreatic duct and parenchymal atrophy, suggesting chronic pancreatitis. An ultrasound-guided biopsy of metastatic muscle deposits in the cervical region was performed.

Histopathology (Fig. 1G, H, I, J, K, L, M) revealed an invasive parathyroid neoplasm arranged in lobules, with tumor cells exhibiting mild nuclear atypia. The parathyroid tumor cells infiltrated skeletal muscle bundles, and vascular invasion (arrowhead) was confirmed on CD31 immunohistochemistry (IHC) and H&E, 100×. Increased mitotic figures were observed, and the Ki-67% proliferative index was 8%. The tumor infiltrated adjacent skeletal muscle bundles with focal vascular invasion but without necrosis or lymphatic or perineural invasion. IHC showed positive tumor cells for PAX8, GATA3, Chromogranin, and BCL2; retained E-cadherin expression; and negative staining for TTF1, Synaptophysin, and D240.

The diagnosis of malignant transformation of PM into PC was established based on the lesion’s local invasion into the surrounding tissues of the trachea, esophagus, and carotid vessels, along with distant metastasis to the cervical lymph nodes and lungs (Supplementary Table 1). CD31 positivity on immunohistochemistry, indicating tumor cells within blood vessels in the pseudocapsule (vascular invasion), and a Ki-67% proliferative index of 8% (Fig. 1) confirmed the diagnosis.

## Treatment

The lesion was infiltrating extensively into the trachea, esophagus, and carotid vessels, making it inoperable. Hence, the patient was treated with cinacalcet and is under follow-up.

## Outcome and follow-up

Three months after, the serum calcium levels were within the normal range. He remains under follow-up for potential disease complications and medication management.

## Discussion

This 67-year-old male presented with left-sided neck swelling and was diagnosed with PHPT (clear cell adenoma). Three years after parathyroidectomy, he developed recurrent left-sided neck swelling. Radiological evaluation revealed mediastinal extension of the lesion without lymph node metastasis. During surgery, the lesion was adherent to the lower common carotid artery. Histological evaluation confirmed a diagnosis of PM.

At a recent presentation, the patient had new-onset neck swelling, abdominal pain, and weight loss. Invasive growth into surrounding tissues, blood vessels, and nerves, along with lymph node involvement and lung metastasis, was observed. Histopathology showed CD31 positivity and a Ki-67% proliferative index of 8%, confirming the diagnosis of PC.

PM is characterized by recurrent or persistent PHPT with multiple benign hyperfunctioning nodules of parathyroid tissue scattered throughout the neck and mediastinum (Schulte *et al.* 2021). It commonly occurs as a secondary phenomenon due to intraoperative seeding from suboptimal gland handling during neck exploration for parathyroid surgery. Histopathologically, PM consists of multiple 1–2 mm non-encapsulated nodules of hypercellular parathyroid tissue, often composed of oxyphil, chief, and transitional cells. Rarely, PM may develop spontaneously due to aberrant embryological development (Lee *et al.* 2001).

Differentiating PC from PM is challenging due to overlapping clinical and morphological features. Schulte *et al.* (2021) demonstrated that PC often exhibits coarse chromatin, infiltrative lesions, and metastasis. While both PC and PM can show loco-regional metastasis, the presence of lymph node and distant metastasis, such as in this patient’s lungs, is diagnostic of PC. Serum calcium and parathormone levels alone do not distinguish PA, PM, or PC (Schulte *et al.* 2021, Hage *et al.* 2012). Histological confirmation of vascular invasion, perineural invasion, and distant metastasis is required for a definitive diagnosis of PC. Most PAs do not recur, while PM tends to recur locally and regionally (Hage *et al.* 2012).

In this patient, the initial diagnosis was PHPT (clear cell adenoma), followed by PM as the first recurrence. The second recurrence, with metastasis and vascular invasion confirmed by CD31 positivity on immunohistochemistry and a Ki-67% proliferative index of 8%, established the diagnosis of PC. The progression from PHPT to PM and subsequently to PC is a novel finding documented in this case.

A Ki-67 index >5% suggests malignancy, with lower indices typical of benign parathyroid lesions. A Ki-67 index >10% indicates aggressive behavior and poor prognosis. Loss of parafibromin is another marker of PC (Erickson & Mete 2018, Erickson *et al.* 2022, Uljanovs *et al.* 2022).

Managing metastatic PC focuses on controlling hypercalcemia and tumor growth (Alberti *et al.* 2022). Surgical resection with negative margins is the cornerstone of treatment. However, due to the tumor’s infiltration into major vessels, the trachea, and the esophagus, surgery was not performed. Hypercalcemia was managed with cinacalcet. The extent of disease does not always correlate with hypercalcemia severity. The lungs are the most common site of metastasis. Systemic therapies for hypercalcemia in malignancies include bone resorption inhibitors such as bisphosphonates and denosumab. Denosumab, a monoclonal antibody against RANKL, is effective in refractory hypercalcemia, particularly in cases resistant to zoledronic acid (Alberti *et al.* 2022).

Calcimimetic drugs such as cinacalcet have shown good results (Roukain *et al.* 2022) and are beneficial in recurrent or inoperable PC cases. Anecdotal reports suggest efficacy with systemic anticancer therapies, including alkylating agents and anthracyclines. Dacarbazine and anthracyclines are the chemotherapy agents of choice for metastatic PC. Targeted therapies involving genomic alterations in PTEN, NH1, KDR, PIK3CA, and TSC242 have been explored. A vaccine containing human and bovine PTH-like immunogenic fragments that induce autoantibody formation against PTH has shown potential in controlling tumor growth and hypercalcemia (Alberti *et al.* 2022). Hypercalcemia is the leading cause of mortality in PC. In this patient, cinacalcet (60 mg/day) led to a reduction in serum calcium levels (Silverberg *et al.* 2007). Prognosis in PC varies. The 5-year and 10-year survival rates range from 75 to 100% and 50–90%, respectively (Mohebati *et al.* 2012).

Prognostic factors include the completeness of initial resection and the presence of metastasis. In this case, the tumor was inoperable and was managed with cinacalcet, which effectively reduced hypercalcemia severity. The documentation of the transition from parathyroid adenoma to PM and finally to PC is unique in this clinical presentation.

## Supplementary materials



## Declaration of interest

The authors declare that there is no conflict of interest that could be perceived as prejudicing the impartiality of the work reported.

## Funding

This work did not receive any specific grant from any funding agency in the public, commercial, or not-for-profit sector.

## Patient consent

Written consent from the patient was obtained to use anonymized images and to publish the case.

## Author contribution statement

CVH drafted the initial manuscript, reviewed the literature, and obtained patient consent. AST gathered the images and edited the manuscript. APR gathered the histopathology images, reviewed, and edited the manuscript. All authors reviewed, revised, and finalized the manuscript.
