# Supplementary material for: Malignant transformation of parathyromatosis to parathyroid carcinoma with invasive growth and distant metastasis
Source: Endocr Oncol. 2025 May 31;5(1):e250024. doi: 10.1530/EO-25-0024 (PMC12131739; doi:10.1530/EO-25-0024)
Supplement: Supplementary file 1 [file supplementary_materials.pdf]

Supplementary Table 1 Clinical, biochemical, imaging, and surgical details of the patient

S No	Clinical Features/Diagnosis	Corrected Calcium mmol/L (N 2.3-2.6)	S.Creat umol/L (N 62-106)	S. Phos mmol/L (N1.1-1.45)	S.PTH mmol/L (N 0.8-5.9)	25OHD nmol/L (N >75	SAP IU/L (N 32-126)	Radiology Investigations	Medical/Surgical Management
**2019**									
FEBRUARY	Evaluation for neck swelling.	2.36	187.45	0.77	11.56	15.5	108.47	1. MRI NECK-6.5x5.6x4.8 cms Lesion left lobe of the thyroid. 2.US ABDOMEN-Few calculi 4x8 mm Left renal calyx.	
MARCH	Surgery for neck swelling	2.83-3.2	236.08	0.5-0.82	11.76			1. Tc-99-MIBI Scan - left inferior parathyroid adenoma,<br>2. chest X-ray posterior-anterior view normal	1. Left hemi thyroidectomy with Left inferior parathyroidectomy 2. HPE-Follicular adenoma thyroid; clear cell parathyroid adenoma-well encapsulated clear cells, no mitosis/atypia or capsular or vascular invasion seen.
APRIL		3.3	179.49	0.97	8.46		185.27		
JUNE		2.36	187.45	0.77	11.56	15.5	108.47		
**2022**									

MAY	Recurrence of neck swelling and weight loss	2.64	172.42	0.77	10.2		100.69	1.US Neck- 1.1x0.8 cms thyroid; 0.9x0.7cms superior pole of thyroid nodule; 2. A 2.6x1.1 cm hypoechoic lesion in the subcutaneous plane on the side of the neck 3. MRI neck - 5.1x8.1x8.5 cms lesion extending superiorly to thyroid cartilage; medially to tracheal cartilage; inferiorly to the brachiocephalic trunk; up to superior mediastinum up to manubrium sterni; multiple small foci in subcutaneous plane at insertion of sternomastoid	1. FNAC of the nodule- a tight cluster of cells with a monomorphic nucleus and moderate cytoplasm. 2. trucut biopsy - parathyroid cells dispersed in sheets, round nuclei, uniform chromatin. Mitosis not seen.

JUNE		3.09-3.67	159.16-185.68	0.87					1. left-sided neck mass dissected off, plastered to internal jugular veins, 2. HPE- multiple unencapsulated nodules of parathyroid tissue of varying size and smooth edges in skeletal tissue, with a diffuse trabecular pattern, polygonal cells with round nuclei with anisocytosis, clear to amphophilic cytoplasm, dark nuclei, mitosis 1-2/HPF. no necrosis, vascular or perineural invasion - PARATHYRAMATOSIS
JULY			167.11						
AUGUST		3.62-3.97	185.68	0.77					
SEPTEMBER			150.31						
NOVEMBER			168						
DECEMBER			194.52						
**2023**									
MARCH			183.91						
SEPTEMBER			74.27						
**2024**									

JANUARY	Recurrence of neck swelling		155.62					US - Abdomen- bilateral renal calculi- right side 9 mm lower calyx; left side 7 mm upper pole	
FEBRUARY			88.42				255	CT-Abdomen- Bilateral renal calculi, changes of chronic pancreatitis with intraductal and parenchymal calcification	
MAY		3.27	153	1.74	35.82	62.8	176	see- figures	Trucut biopsy done - see figures
SEPTEMBER		2.45	162						

Abbreviations: Cor. Calcium – Serum Albumin Corrected calcium; S. Phos. – Serum phosphorous; S.PTH – Serum Parathormone; 25 OHD- 25 Hydroxy vitamin D; SAP – Serum alkaline phosphatase; MRI- Magnetic resonance Imaging; US- Ultrasound; HPE- Histopathology Examination.
